# Long-term results of therapeutic local anesthesia (neural therapy) in 280 referred refractory chronic pain patients

**DOI:** 10.1186/s12906-015-0735-z

**Published:** 2015-06-27

**Authors:** Simon Egli, Mirjam Pfister, Sabina M. Ludin, Katia Puente de la Vega, André Busato, Lorenz Fischer

**Affiliations:** Medical Faculty of the University of Bern, Bern, Switzerland; Department of Neural Therapy, IKOM, University of Bern, Inselspital, PH4, CH-3010 Bern, Switzerland; Formerly Professor of Health Services Research, Institute of Social and Preventive Medicine, University of Bern, Bern, Switzerland; Institute of General Practice, University of Zürich, Zürich, Switzerland

**Keywords:** Chronic pain, Treatment resistance, Local anesthetics, Neural Therapy, Neurophysiology

## Abstract

**Background:**

Can the application of local anesthetics (Neural Therapy, NT) alone durably improve pain symptoms in referred patients with chronic and refractory pain?

If the application of local anesthetics does lead to an improvement that far exceeds the duration of action of local anesthetics, we will postulate that a vicious circle of pain in the reflex arcs has been disrupted (hypothesis).

**Methods:**

Case series design. We exclusively used procaine or lidocaine. The inclusion criteria were severe pain and chronic duration of more than three months, pain unresponsive to conventional medical measures, written referral from physicians or doctors of chiropractic explicitly to NT. Patients with improvement of pain who started on additional therapy during the study period for a reason other than pain were excluded in order to avoid a potential bias. Treatment success was measured after one year follow-up using the outcome measures of pain and analgesics intake.

**Results:**

280 chronic pain patients were included; the most common reason for referral was back pain. The average number of consultations per patient was 9.2 in the first year (median 8.0). After one year, in 60 patients pain was unchanged, 52 patients reported a slight improvement, 126 were considerably better, and 41 pain-free. At the same time, 74.1 % of the patients who took analgesics before starting NT needed less or no more analgesics at all. No adverse effects or complications were observed.

**Conclusions:**

The good long-term results of the targeted therapeutic local anesthesia (NT) in the most problematic group of chronic pain patients (unresponsive to all evidence based conventional treatment options) indicate that a vicious circle has been broken. The specific contribution of the intervention to these results cannot be determined. The low costs of local anesthetics, the small number of consultations needed, the reduced intake of analgesics, and the lack of adverse effects also suggest the practicality and cost-effectiveness of this kind of treatment. Controlled trials to evaluate the true effect of NT are needed.

## Background

### Background and objective

The number of chronic pain syndromes is rapidly increasing [1, 2]. Moreover, chronic pain has an enormous socio-economic impact [3]. The hopes that have in the past years been placed on efficient medical drug therapies causing very few side effects have been disappointed in parts. This is why non-medical treatment options have been sought after, too. A logical option to consider is the regulating therapy using local anesthetics (Neural Therapy, NT), which is based on both experience [4, 5] and the findings of modern pain physiology [6, 7]. A national cross-sectional survey that was conducted among 300 randomly selected primary care physicians in Germany attests to the great importance of NT in terms of application frequency and subjective perception of efficacy [8].

The present case series was undertaken to answer the question if the application of NT alone (i.e., without using any additional measures) can produce long-term improvement of the pain symptoms in referred patients with chronic and so far refractory pain. A case series design was used to investigate the level of pain relief, the consumption of pain medication as well as safety issues.

At the same time, we tested the following hypothesis, postulated on the basis of the interpretation of findings from modern neurophysiology of pain: A vicious circle in the pain process can be disrupted by injecting local anesthetics into suitable sites of the body, which is reflected by the fact that the effect lasted much longer than could be expected from the duration of action of the local anesthetic. At the same time, we accept that specific and non-specific effects of treatment cannot be clearly discriminated.

### Definition of neural therapy

NT is a treatment modality using injections with local anesthetics for diagnosis and therapy (indications include functional disorders, inflammatory diseases and acute and chronic pain). The real purpose, though, is not to achieve local anesthesia (except for diagnosis). The generation of targeted stimuli (through the needle) and the selective extinction of other stimuli (through the local anesthetic) affect both the organization of the nervous system and tissue perfusion, thereby disrupting positive feedback actions (vicious circle) in the pain cycle. This treatment modality utilizes the regulatory mechanisms and plastic properties of the nervous system, mainly on two levels: first, via segmental reflectory processes [9–11], and second, via the so called interference field (irritation zone), which may initiate and/or maintain pain and inflammation, regardless of the involved segment [4, 5, 9–16]. The neurophysiological rationale and the mechanisms of action will be outlined in the discussion section.

## Methods

### Design

A case series design was used including patients from a University practice specializing in NT (Professor of Neural Therapy, University of Bern). All patients that met the inclusion criteria and had been referred explicitly to NT between January 1, 2000 and December 31, 2007, were included in the retrospective analysis. Each patient was followed over a period of 12 months. The local ethics committee concluded that no special approval was needed for the following reasons, and therefore, granted permission for us to access patients’ data for the purposes of our study: Not only the referral explicitly to NT, but the treatment, too, was completely independent from the retrospective analysis; written informed consent was obtained from all patients; and the application of an accepted treatment was by definition in full compliance with the statements in the Helsinki Declaration. Therefore, the study passed the review process at the University of Bern to be accepted as doctoral thesis.

### Patients

#### Inclusion criteria

- Patients with pain and written referral explicitly to NT from physicians or doctors of chiropractic
- Chronic condition lasting more than 3 months
- Treatment-resistant pain, i.e., pain persisting after all other evidence-based (conventional medical) measures compatible with the diagnosis (especially pain medication) have failed.
- Only patients were included whose pain was at least severe,^1^ regardless of their pain medication (example: If pain medication had reduced a patient’s pain from very severe to mild levels at recruitment, this patient was excluded since his pain was neither treatment-resistant nor was there any drug intolerance).

#### Exclusion criteria / drop-out

- Conventional medical treatment options (in accordance with the diagnosis) have not been fully exhausted at the beginning of NT.
- To avoid a potential bias, patients with improvement of pain who started on another therapy during the one-year treatment period for a reason other than pain but with the potential to influence the pain level in a positive way (e.g. rigorous diet, psychotherapy, etc.) were excluded from the analysis.
- Discontinuation of NT due to accident, new onset of a serious disease, moving house, etc.

### Intervention

Exclusive diagnostic and therapeutic application of the local anesthetics procaine or lidocaine in terms of NT (see definition).

### Data acquisition

During the *first consultation* data were collected on age, sex, duration of pain problem, diagnosis, severity of pain, secondary diagnoses, and both the outcomes of previous therapies and the medication documented. For each individual consultation, interventions with local anesthetics, change in pain, medication use, adverse effects and complications were recorded over a *12-month study period*. At the *final visit (12 months after starting NT)* the patients were asked to indicate their current level of pain, and both the number of consultations during the past 12 months and the current medication was documented.

In order to grade the severity of pain (see inclusion criteria) and examine the change in pain a deliberate choice was made to forego the use of a visual analog scale in favor of MacNab’s criteria, which for the purpose of the present case series seemed to be more appropriate (modified according to Schmid [17]). Regarding the change in pain after one year the results were as follows: I: no pain, II: considerable improvement (more than 50 %), III: slight improvement (less than 50 % of the initial pain at the end of the follow-up period), IV: no change, V: worsening. Additionally, patients were not only asked to selectively rate their pain level, but also the average monthly pain level in the month before beginning NT and in the twelfth month. Again, the simple MacNab criteria appeared to us to be more practical than the visual analog scale.

Patients with a fluctuating course who reported improvements with NT, but rated their level of pain after one year as being no different from the pain intensity before the beginning of NT, were assigned to the “no improvement” category. In the case of patients with multiple pain problems only the primary diagnosis (cause of referral) was evaluated.

For purposes of documenting the medication use after treatment with NT (after one year) the following categories were used: 1. more medication than before starting treatment, 2. less medication, 3. no change in medication intake, 4. no medication taken before and after treatment. Only pain-relieving and pain-modulating drugs were considered.

The case series did not include patients who had not fully exhausted either conventional medical treatments previously or had not been resistant to treatment before beginning NT. Patients were not excluded, though, if NT was prematurely discontinued due to lack of success, but were assigned to the “no improvement” category (two patients).

The diagnoses were recorded according to the ICD-10 Code and then subdivided into four large pain-related diagnostic groups: 1. disorders of the spine and back, 2. disorders of other parts of the motor system, 3. headaches, 4. other pains.

The outcome of NT interventions were summarized in a report and communicated to the referring physician.

### Statistical procedures

Fisher’s exact test was used to statistically identify differences in the frequency between groups. With continuous and discrete data (age, number of treatments, etc.), the Wilcoxon or the Kruskal-Wallis test was used to determine the magnitude of difference between groups. Monotonic associations between some of the variables were identified using Spearman’s rank correlation coefficient. A *p* value of <0.05 was used to denote statistical significance.

## Results

### Demographic data and the Patients’ general state of health

In the above-mentioned period a total of 361 chronic pain patients had a written referral for NT. 59 patients were not included since their pain levels did not exceed categories 1–3 (see inclusion criteria). Seven patients could not be enrolled in the case series because they had started another treatment simultaneously with the beginning of NT. To avoid another potential bias, eleven patients with improvement of pain were later excluded because they began another treatment for a reason other than pain during the one-year study period, potentially influencing the pain level in a positive way (e.g. rigorous diet, psychotherapy, etc.). Another four patients were excluded because of external factors (accident, moving home, etc.).

Finally, 280 patients were included in the case series. All patients suffered from chronic pain which had so far been treated with no success and may thus be regarded as unresponsive to treatment. All conventional treatment options had been fully exhausted in accordance with the respective diagnosis. All patients had previously received medical treatment; in 31 % of the patients the pharmacologic treatment approach of pain had been stopped before starting NT, one reason being a lack of effect, another the occurrence of adverse effects. More than two thirds of the patients had previously undergone physical therapy, physiotherapy, osteopathy or chirotherapy, and 25 % of the patients had also tried acupuncture. All pain conditions were resistant to these procedures as well.

176 women and 104 men were included; the average age of the females was 50.1 years (SD: 15.1; range: 14–84) and of the males 50.9 years (SD: 14.4; range: 11–90) (Table 1).

**Table 1:** Distribution by age and sex

Sex	n	Age (years)		
		Mean (SD)	Median	Range
Female	176	50.1 (15.1)	51	14–84
Male	104	50.9 (14.4)	51	11–90
Total	280	50.4 (14.9)	51	11–90

### Specialties of referring physicians

All patients were referred by physicians from a broad spectrum of professional disciplines (Table 2). 36 of the patients had referrals from various specialist clinics of a university hospital.

**Table 2:** Distribution of referring doctors

Referring physicians by specialty	Frequency of referral
General Medicine	147
Chiropractic	38
Internal Medicine	30
Rheumatology	16
Orthopedic Surgery	10
Otorhinolaryngology	8
Ophthalmology	8
Hand Surgery	5
Pediatrics	4
General Surgery	3
Anesthesia (Pain Clinics)	2
Gynecology	2
Psychiatry	2
Urology	2
Neurology	1
Insurance Medicine (casualty insurer SUVA^a^)	1
Dentistry	1

Various physicians referred several patients. The 5 physicians who most frequently referred patients with treatment-resistant pain sent the following number of patients during the enrollment period: 27, 21, 15, 13 and 8, respectively

^a^ SUVA (Swiss National Accident Insurance Fund) is the largest provider of accident insurance in Switzerland

### Symptoms and diagnoses

More than two thirds of the patients (69.6 %) suffered from treatment-resistant pain disorders of the spine and of other parts of the motor system. Every eighth patient (12.2 %) complained of headache (Table 3).

**Table 3:** Diagnosis codes

Diagnosis (ICD 10)	Frequency	%
1. Disorders of the spine and back	155	55.3
2. Other disorders of the motor system		
Arthropathies	18	6.4
Soft tissue disorders	16	5.7
Osteopathies	4	1.4
Chondropathies	2	0.7
Systemic connective tissue disorders	1	0.4
Total	41	14.6
3. Headaches		
Atypical facial pain	13	4.7
Chronic post-traumatic headache	5	1.8
Cluster headache	5	1.8
Other headaches	5	1.8
Migraine	4	1.4
Trigeminal neuralgia	2	0.7
Total	34	12.2
4. Other pain		
Diseases of the eye	7	2.5
Diseases of the genitourinary system, male	5	1.8
Diseases of the genitourinary system, female	3	1.1
Diseases of the digestive system	3	1.1
Other diseases	32	11.4
Total	50	17.9
All groups	280	100

### Duration of illness

The mean duration of illness in all patients before starting NT was 64 months, i.e., more than five years. The distribution of duration of illness was skewed, meaning that 50 % of all patients had been ill for less than 36 months and 4 patients for more than 360 months. Table 4 shows the mean (incl. SD) and median duration of illness of the four most important diagnostic groups. There was a statistically confirmed difference in the duration of illness between these four diagnostic groups (*p* = 0.04), in that patients afflicted with headache and facial pain showed a longer duration of illness before their referral than the other three groups (Table 4). An analysis of the duration of illness in relation to five age categories (0–20, 21–40, 41–60, 61–80, >80 years of age) did not reveal any significant differences between the age groups (*p =* 0.14). Also, there was no difference between female and male patients (*p* = 0.41).

**Table 4:** Duration of Illness before the beginning of neural therapy (in months)

Diagnosis code	Patients (n)	Duration of illness (months)	
		Mean (SD)	Median
Disorders of the spine and back	155	64.65 (91.59)	36
Other disorders of the motor system	41	43.98 (55.75)	24
Headache	34	106.50 (130.30)	54
Other pain	50	49.50 (53.54)	24
Total	280	64.00 (88.91)	36

### Number of treatments

The number of treatment sessions utilized by patients in the first year varied between one and 40, the average number being 9.16 (SD: 5.69), and the median number 8.0. There was a statistically confirmed difference in the number of treatments between diagnostic groups (*p* = 0.01). In the first year, patients suffering from painful musculoskeletal disorders affecting the spine and back needed more consultations than the other three groups (Table 5). There were no significant differences in the number of consultations in relation to age groups (*p* = 0.11), but female patients required significantly more (i.e., 10.6) consultations (*p* < 0.01) than male patients (7.6 consultations).

**Table 5:** Number of treatments in the first year

Diagnosis code	Patients (n)	Treatments	
		Mean (SD)	Median
Disorders of the spine and back	155	10.00 (5.97)	8.00
Other disorders of the motor system	41	7.07 (4.95)	5.00
Headache	34	8.44 (5.12)	8.50
Other pain	50	8.78 (5.31)	9.00
Total	280	9.16 (5.69)	8.00

Correlation coefficients between age, duration of illness and number of consultations and their corresponding *p* values are listed in Table 6. The correlation coefficients reveal a weak and non-significant linear association between age and duration of illness and a weak, but significant association between the age and the number of consultations.

**Table 6:** Correlation coefficients between age, duration of illness and number of consultations

	Age		Duration of illness	
	correlation coefficient	*p* value ^*^	correlation coefficient	*p* value
Duration of illness	0.11	0.06		
Consultations	0.14	0.01	0.06	0.28

^*^ *p* value, i.e., the statistical test for a correlation coefficient ≠ 0

### Change in pain

Treatment success from the patients’ perspective is shown in Table 7. One patient felt that his pain had worsened, and 60 patients reported that their symptoms were unchanged. Rating the success of their treatment after one year, 52 patients saw a slight and 126 patients a considerable improvement. 41 patients were pain-free after one year. Treatment success was not significantly different between the diagnostic groups (*p* = 0.14).

**Table 7:** Diagnostic groups versus change in pain after one year

	Disorders of the spine and back		Other disorders of the motor system		Headaches		Other pain		Total	
	n	%	n	%	n	%	n	%	n	%
Patients	155	(100)	41	(100)	34	(100)	50	(100)	280	(100)
Change in symptoms										
Worsening	0	(0.0)	0	(0.0)	0	(0.0)	1	(2.0)	1	(0.4)
No change	31	(20.0)	11	(26.8)	10	(29.4)	8	(16.0)	60	(21.4)
Slight improvement	32	(20.7)	5	(12.2)	5	(14.7)	10	(20.0)	52	(18.6)
Marked improvement	77	(49.7)	15	(36.6)	12	(35.3)	22	(44.0)	126	(45)
No pain	15	(9.7)	10	(24.4)	7	(20.6)	9	(18.0)	41	(14.6)

157 patients (56 %) underwent local/segmental therapy, 5 patients (2 %) received interference field therapy, and 118 patients (42 %) a combined (local/segmental plus interference field) treatment (Table 8). A comparison of change of pain between a purely local/segmental and a combined therapy did not reveal any statistically confirmed difference (*p* value Fisher test = 0.28). Due to the small number of patients, interference field therapy alone was not included in the statistical calculations (Table 8).

**Table 8:** Treatment modality vs. change in pain after one year

	Local/segmental		Interference field		Combined	
	n	%^a^	n	%^a^	n	%^a^
Patients	157	(100)	5	(100)	118	(100)
Change in symptoms						
Worsening	1	(0.6)	0	(0.0)	0	(0.0)
No change	34	(21.7)	0	(0.0)	26	(22.0)
Slight improvement	29	(18.5)	3	(60.0)	20	(17.0)
Marked improvement	65	(41.4)	1	(20.0)	60	(50.9)
No pain	28	(17.8)	1	(20.0)	12	(10.2)

^a^ percentage of the respective treatment modality

### Consumption of pain medication

Table 9 displays the consumption of pain medication after one year as compared to the consumption before NT, separately listed for the group of patients with successful NT (n = 219; slight improvement to freedom from symptoms) and for the group of patients with no treatment success (n = 61; no change or worsening). Successfully treated patients needed significantly less pain medication (p < 0.01).

**Table 9:** Medication intake and change in symptoms

	Treatment effect^a^			
Medication intake	No success		Success	
	n	%^b^	n	%^b^
No medication	18	(29.5)	69	(31.5)
No change in medication intake	40	(65.6)	10	(4.6)
Reduced use of (or no more need for) medication	3	(4.9)	140	(63.9)
Increased use of medication	0	(0)	0	(0)
Total	61	(100)	219	(100)

^a^ according to the patients’ self-assessment

^b^ percentage within this category

Eighty-seven patients did not take any pain medication, neither when they were included nor after they started NT. However, these patients had – at some earlier point during the course of their disease – also received one or more pain-relieving drugs prior to NT, but had discontinued their medication because of lack of effect or intolerable adverse effects. After one year none of these patients used any pain medications.

In 50 patients (25.9 % of the patients taking pain relievers at the beginning of NT) pain medication consumption was unchanged, and 143 patients (74.1 %) used less or no more pain medication at all after the period under study.

### Observation of adverse effects or complications

No adverse effects or complications occurred except minor, spontaneously resolving hematoma and mild dizziness lasting up to 15 min following treatment, which in patients with normal blood pressure was assessed as the known systemic procaine effect and simultaneous, mild vasovagal reaction. There was no case where an adverse effect would have required drug treatment or any other intervention.

## Discussion

### Neurophysiology and mechanisms of action

**Fig. 1:**
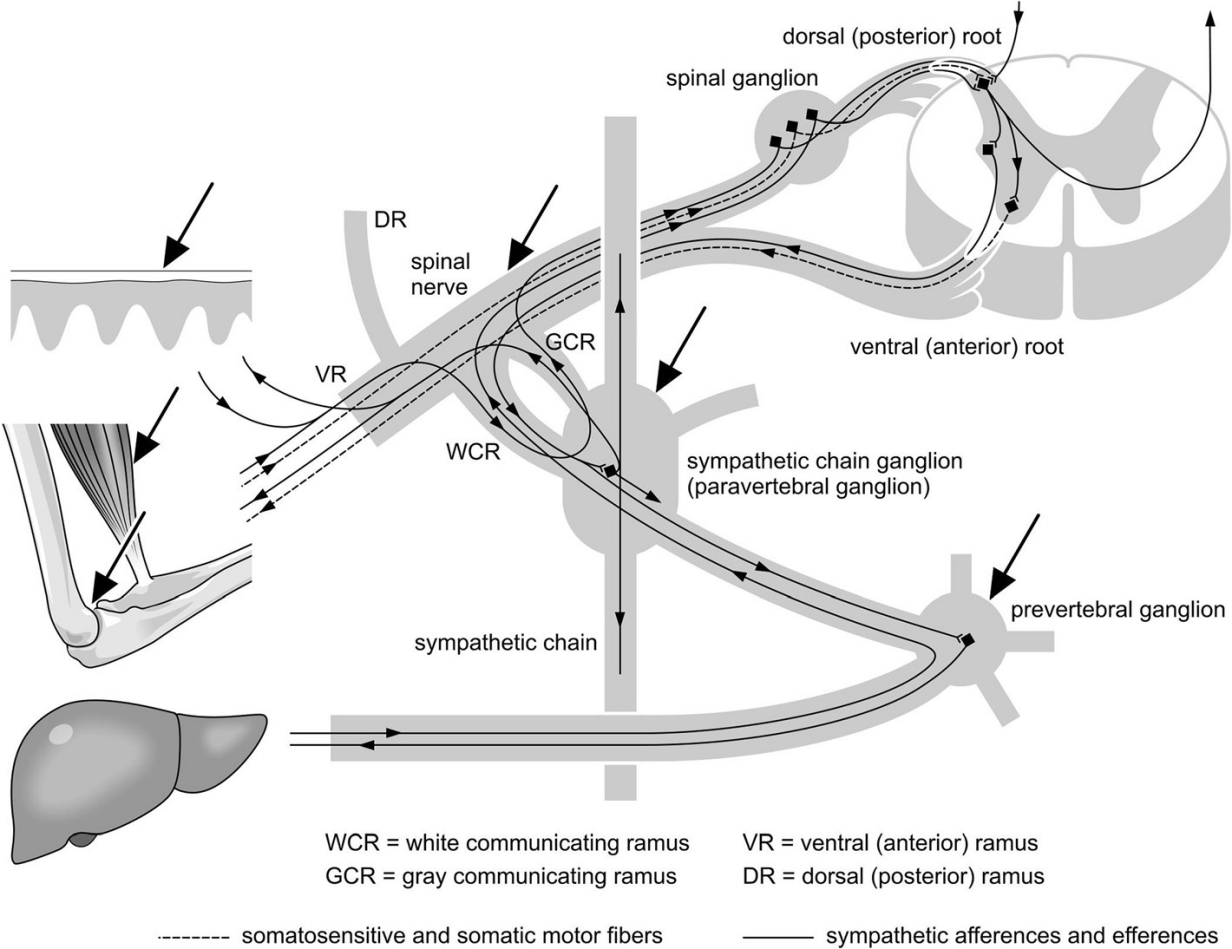
Reflectory connections between skin, muscle and internal organ: a simplified schematic diagram. The arrows indicate possible sites of injection with local anesthetics. The injections can be combined

Nociceptive processes cause a reflex response evoked by cutivisceral, viscero-cutaneous, viscero-somatic motor, etc. reflex pathways. This reflex response, which is largely mediated by sympathetic nerves, involves changes in blood flow, increased skin turgor, hyperalgesia in localized areas of skin, dysregulation of the organ at the corresponding metameric level, as well as increased muscle tone [18–20]. Nociceptive afferents converge in the dorsal horn of the spinal cord. The information from this area is then diverged: to the sympathetic nervous system, to the skeletal muscles, and to the brain, all at the same time (Fig. 1) [6, 7, 21].

Analogous to mathematical chaos theory these connections create a vicious circle (positive feedback, iteration) of pain [20]. This vicious circle is reinforced because under pathological conditions efferent (!) sympathetic nerves in the periphery are able to relay to nociceptive afferents in a kind of short circuit, leading to a coupling between sympathetic and afferent neurons [7, 22–24]. Sympathetic-afferent coupling and also neuroplastic changes [25] within the spinal cord and in the brain produce additional multiple iterations of the vicious circle of pain.

The sympathetic nervous system can induce a neurogenic inflammation via vasodilation and plasma extravasation and the release of pro-inflammatory neuropeptides from its own nerve fibers [26–31]. This inflammation decreases the response threshold of nociceptors and simultaneously recruits quiescent or “silent” nociceptors from the neighborhood. Thus peripheral sensitization occurs [22], which reinforces the vicious circle even further.

Tracey [32] describes an “inflammatory reflex of the autonomic nervous system,” reflexively adjusting the inflammatory and immune responses of the human body.

As early as 1924 Ricker [33] was able to demonstrate in an animal model that even injuries to the sympathetic nervous system or pathological irritations which occurred a long time ago would become engrammatically stored. Every new (physiological) stimulus to such a system causes a pathological (excessive) response. It seems that the sympathetic nervous system has a kind of “memory” for pathological stimuli.

Using local anesthetics (NT) these different levels can be accessed directly and logically: by applying an impulse (needle prick) and by disrupting a vicious circle (local anesthetic). Repeated application can lead to the “extinction” of the engrammatically stored pathological irritability of the sympathetic nervous system and to the restoration of normal tissue perfusion [9, 20, 34]. The local anesthetic can disrupt the escalating vicious circle of nociceptor activity – sympathetic excitation – circulation disturbance – neurogenic inflammation – muscle hardening, etc. in different sites at the same time (Figure 1). In this way several interrelated and, through positive feedback loops, ever-increasing reflex arcs become disrupted.

Moreover, Cassuto [26] was able to show that repeated application of local anesthetics can also directly reduce neurogenic inflammation.

In addition, needle prick and local anesthetic can produce a favorable effect on the control of synaptic input to the neurons in the dorsal horn of the spinal cord [35].

### Interpretation of data

Reflecting the real-life setting of our University NT practice, our case series included the most difficult category of patients with chronic pain, i.e. chronic pain unresponsive to all evidence-based, conventional medical measures and various complementary treatments over a long period, who had been referred explicitly to NT. For these reasons randomization of a part of these patients in a control group without NT was not possible. Upon closer consideration, the fact that all previous therapies had failed can serve as a kind of comparison (albeit with some time lag) between conventional (incl. complementary) medical treatments and NT, but cannot, of course, replace a real control group. However, there is a probability of a spontaneous effect of pain regression to the mean because the pain level of our patients was severe. Therefore, it would be expected that some patients by chance would find their pain reduced over the study period. Without a control group it is therefore difficult to assess what amount of pain reduction is due to the treatment and what amount due to the potential regression to the mean. Nevertheless, it is fair to say that the mean duration of pain illness without regression before NT was more than five years. After this long period, a significant improvement without therapy seems improbable.

Despite the severity of the pain disorder and the long duration of illness prior to NT, patients only needed an average of 9.2 consultations within a period of one year.

In the long term only one fifth of our patients remained resistant to NT (as to conventional medical treatment options before). Another fifth experienced a slight improvement, and three fifths a marked improvement of pain or even freedom of pain. These results are supported by a Health Technology Assessment report [4] and other studies [36–38]. Furthermore, NT, which has been empirically developed in normal, everyday clinical practice, is in line with modern knowledge of the neurophysiology of pain and neurogenic inflammation [7, 26, 29, 32, 34, 39–42].

Combined injections of local anesthetics (NT) primarily affect the efferent and afferent fibers of the peripheral spinal reflex arc, largely via the sympathetic nervous system involved in the pain processes. Due to the connection of the central reflex arcs in the brain stem and in the cortex with neurons within the spinal cord it seems likely that they are indirectly affected by NT. On the other hand, these central reflex arcs are also subject to other influences, such as emotions (for example, the doctor-patient relation), especially in the presence of the respective neuroplastic changes. For this very reason we cannot determine the exact size of the specific effect of NT with our present results. However, the majority of our patients had received interventions within a similar doctor-patient setting (physiotherapy, acupuncture, psychotherapy, manual therapy, interventional pain management) prior to their enrollment in this case series, and still had been unresponsive to treatment. This is why we suppose that a part of the effect of NT may be specific.

The second outcome measure was the consumption of pain-relieving drugs: out of the 193 patients under pain medication three quarters took less pain medication or none at all after one year. These findings are noteworthy for their cost-effectiveness implications. In our cross-sectional studies [38, 43] commissioned by the Swiss Federal Office of Public Health (BAG) we also noted better cost-effectiveness implications in primary care providers who had incorporated NT in their practice (compared to primary care providers offering conventional medical treatment alone). The same results we saw filing an application to the Swiss Federal Office of Public Health [5]: we were able to compile the following data from the Association of Swiss Health Insurance Companies (SantéSuisse): primary care providers offering integrative NT were compared to those providing conventional medicine alone. Both total costs and medication costs (average costs per year per patient) were significantly lower in the NT group.

### Limitations

Although the present case series was conducted under circumstances similar to those found in daily practice and its results can be transferred into the clinical and practice setting, there are still limitations. As it was – in this real-life setting with patients referred explicitly to NT – not possible to opt for a controlled study design, the exact size of the specific effect of NT cannot be determined in this case series. Although the chance of a significant spontaneous improvement without therapy in our patients with refractory chronic pain over a long period was little, this possibility cannot be ignored. Being designed as a case series and not a multicenter trial makes it difficult to generalize the findings. Moreover, general quality of life and activities of daily living as well as secondary diagnosis were recorded in the course of history taking, but not included in the analysis of data.

## Conclusions

Chronic pain, especially back pain, is extremely common. Treatment-resistant pain syndromes or adverse effects of medical treatment demand other treatment options. Both the present results for the outcome measures pain and medication use are in line with the results of other studies and demonstrate that therapeutic local anesthesia (NT) is a good treatment option. This is also reflected in the fact that many of the referring physicians were pain specialists or specialists in musculoskeletal disorders, and that every eighth referral was made by university hospitals or their outpatient departments. The good long-term outcomes achieved in just a few visits – such as medication reduction and virtually no side effects – are interesting from an economic perspective as well. As discussed above and mentioned under limitations, we cannot determine the exact size of the specific effect of NT in this uncontrolled case series. It would be, therefore, worthwhile to carry out a controlled trial to evaluate the true effect of NT.

Nevertheless, the results of our study can be logically explained by mechanisms of action that are based on modern neurophysiological concepts of pain. In addition, the fact that in most patients the effect persisted much longer than was to be expected from the duration of action of the local anesthetic also supports our initial hypothesis that the injection of local anesthetics at suitable sites can disrupt the vicious circle of pain (multiple reflex arcs maintaining each other through positive feedback) and thus initiate a reorganization (self-organization) of the pain-processing systems. The results of further research into the mechanisms of action on the one hand and the outcome of future randomized controlled trials on the other will be eagerly awaited.

## Footnotes

1 Categories 4–6 (1 = no pain, 2 = mild pain, 3 = moderate pain, 4 = severe pain, 5 = very severe pain, 6 = unbearable pain)
